# Comprehensive evaluation for the one-pot biosynthesis of butyl acetate by using microbial mono- and co-cultures

**DOI:** 10.1186/s13068-021-02053-2

**Published:** 2021-10-16

**Authors:** Yang Lv, Yujia Jiang, Jiasheng Lu, Hao Gao, Weiliang Dong, Jie Zhou, Wenming Zhang, Fengxue Xin, Min Jiang

**Affiliations:** 1 State Key Laboratory of Materials-Oriented Chemical Engineering, College of Biotechnology and Pharmaceutical Engineering, Nanjing Tech University, Puzhu South Road 30#, Nanjing, 211800 People’s Republic of China; 2 Jiangsu National Synergetic Innovation Center for Advanced Materials, Nanjing Tech University, Nanjing, 211800 People’s Republic of China

**Keywords:** Butyl acetate, Microbial mono-culture, Microbial co-culture system, Immobilization technology

## Abstract

**Background:**

Butyl acetate has shown wide applications in food, cosmetics, medicine, and biofuel sectors. These short-chain fatty acid esters can be produced by either chemical or biological synthetic process with corresponding alcohols and acids. Currently, biosynthesis of short chain fatty acid esters, such as butyl butyrate, through microbial fermentation systems has been achieved; however, few studies regarding biosynthesis of butyl acetate were reported.

**Results:**

In this study, three proof-of-principle strategies for the one-pot butyl acetate production from glucose through microbial fermentation were designed and evaluated. (1) 7.3 g/L of butyl acetate was synthesized by butanol-producing *Clostridium acetobutylicum* NJ4 with the supplementation of exogenous acetic acid; (2) With the addition of butanol, 5.76 g/L of butyl acetate can be synthesized by acetate-producing *Actinobacillus succinogenes*130z (Δ*pflA*); (3) Microbial co-culture of *C. acetobutylicum* NJ4 and *A. succinogenes*130z (Δ*pflA*) can directly produce 2.2 g/L of butyl acetate from glucose by using microbial co-culture system with the elimination of precursors. Through the further immobilization of *A. succinogenes*130z (Δ*pflA*), butyl acetate production was improved to 2.86 g/L.

**Conclusion:**

Different microbial mono- and co-culture systems for butyl acetate biosynthesis were successfully constructed. These strategies may be extended to the biosynthesis of a wide range of esters, especially to some longer chain ones.

**Supplementary Information:**

The online version contains supplementary material available at 10.1186/s13068-021-02053-2.

## Background

Short-chain fatty acid esters are a group of high value-added chemicals derived from alcohols and carboxylic acids [1–3]. These esters naturally exist in some flowers and fruits, which have been widely applied in food, cosmetics, and medicine industries [4–6]. Butyl acetate with a sweet smell of banana is a typical short-chain fatty acid ester, which can not only be used as a fruit flavoring in foods, such as candy, ice cream, and baked goods, but also a high-boiling solvent with moderate polarity [7–9]. In addition, it can also be used as a potential biodiesel additive [10]. When butyl acetate is mixed with biodiesel, the combustion heat and cetane number of biodiesel are not affected. Furthermore, the emission of soot and greenhouse gases is significantly reduced [11]. Meanwhile, owning to its lower freezing point, the addition of butyl acetate will improve the fluidity of biodiesel at low temperature, which indicates promising potentials in aviation sectors [12].

Traditionally, butyl acetate can be synthesized by the Fischer esterification of acetic acid and butanol with the presence of catalytic sulfuric acid under high temperature [13–15]. However, some disadvantages occurred in this chemical conversion process, such as the strong corrosiveness of catalysts, by-products generation, and environmental pollution et al. [16]. Alternatively, biological conversion of butanol and acetic acid to butyl acetate under the catalysis of lipases has attracted more attentions owning to its mild reaction conditions and environmentally friendly properties, which also provides an energy-saving route for the esters production [17–20].

Actually, the biological synthesis of some short-chain fatty acid esters, such as butyl butyrate has been achieved through microbial fermentation process [1, 15, 19]. Currently, two strategies were mainly adopted for short-chain fatty acid esters synthesis: microbial monoculture and co-culture fermentation [15, 17]. For the microbial monoculture fermentation strategy, microbe can synthesize one precursor, such as acid, and the other precursor, such as alcohol, can be exogenously supplemented [15, 20]. With the catalysis of exogenous lipases, acid and alcohol can be converted to their relevant short-chain fatty acid esters [1]. Under the guidance of this principle, 22.4 g/L of butyl butyrate can be directly synthesized by butanol-producing *Clostridium* sp. strain BOH3 with the exogenous supplementation of 7.9 g/L of butyrate in the fed-batch fermentation process [20]. Furthermore, when 10 g/L of butanol was added into the butyric acid-producing fermentation broth by using *C. tyrobutyricum*, 34.7 g/L of butyl butyrate can be synthesized, representing the highest butyl butyrate production through microbial fermentation process [15]. Although high titer of butyl butyrate can be obtained through microbial monoculture fermentation process, high amounts of precursors should be supplemented, which will increase the cost. Alternatively, microbial co-culture strategy offers one promising way, as strain members can be specifically designed to synthesize alcohol and acid, respectively, with the elimination of exogenous addition of acid or alcohol [1, 17]. For example, one clostridial consortium composed of butanol-producing *C. beijerinckii* and butyrate-producing *C. tyrobutyricum* has been designed, which could directly produce 5.1 g/L of butyl butyrate from glucose without the addition of any exogenous precursors [1]. Furthermore, a cognate “diamond-shaped” *Escherichia coli* consortium was also metabolically constructed, which was capable of producing 7.2 g/L of butyl butyrate, resenting the highest butyl butyrate production by using microbial co-culture system [17].

Although butyl butyrate production through the microbial fermentation process has been comprehensively studied, there are only few reports regarding the butyl acetate production [17, 18, 21]. Previously, we have genetically constructed *Actinobacillus succinogenes*130z (Δ*pflA*), which could efficiently produce acetic acid from glucose [22]. Furthermore, one butanol hyper producer of *C. acetobutylicum* NJ4 was isolated and stored by our lab [23, 24]. Accordingly, three proof-of-principle strategies for butyl acetate production were comprehensively evaluated, including *A. succinogenes*130z (Δ*pflA*) fermentation with the exogenous supplementation of butanol, *C. acetobutylicum* NJ4 fermentation with the exogenous supplementation of acetic acid, and microbial co-culture fermentation composed of *A. succinogenes*130z (Δ*pflA*) and *C. acetobutylicum* NJ4. The fermentation conditions of these three systems were optimized, and the relationship between strain members in the microbial co-culture system was also analyzed. Finally, the final titer of butyl acetate by using microbial co-culture system was improved by immobilization technology.

## Results

### Biosynthesis of butyl acetate by using microbial monoculture of *C. acetobutylicum* NJ4 with the supplementation of exogenous acetic acid

As stated in our previous studies, *C. acetobutylicum* NJ4 is a hyper butanol producer, which shows great potential for butyl acetate synthesis through the supplementation of acetic acid [23, 25, 26]. It is also known that lipases can directly catalyze acetic acid and butanol to butyl acetate, and the in situ extraction of butyl acetate could further improve the butyl acetate production and maintain catalytic activities of lipase rather than hydrolytic activities [15, 20]. To obtain high butyl acetate production by using microbial monoculture of *C. acetobutylicum* NJ4, pH, supplemented acetic acid concentration, and acetic acid addition time were comprehensively investigated (100 U/mL lipases and 50% dodecane). The optimization process by using “one factor at a time” strategy was first adopted, and the optimized conditions were as follows: fermentation pH at 5.5, acetic acid concentration at 15 g/L, and acetic acid addition time at 120 h (Additional file 1: Figures S1–S3).

**Fig. 1:**
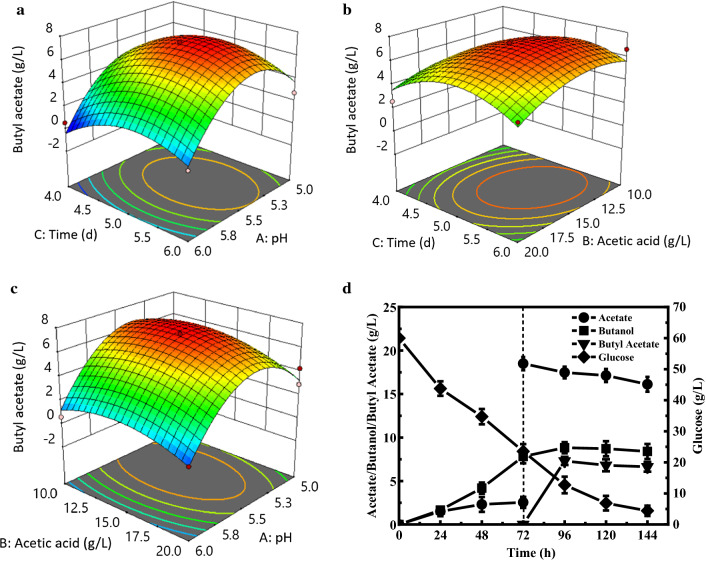
3D response surface curves of the interactive effects including pH, adding time, and concentration of acetic acid on butyl acetate production. **A** Acetic acid addition time and medium pH of *C. acetobutylicum* NJ4 at fixed level of acetic acid concentration. **B** Acetic acid addition time and acetic acid concentration of *C. acetobutylicum* NJ4 at fixed level of pH. **C** Acetic acid concentration and pH of *C. acetobutylicum* NJ4 at fixed level of acetic acid addition time. **D** Fermentation profiles of *C. acetobutylicum* NJ4 under optimal conditions

Response surface methodology (RSM) was further performed with 17 groups of experiments including five replications of the central point, where *Y* is butyl acetate production (g/L), *X*_1_ represents pH values, *X*_2_ represents the supplemented acetic acid concentration (g/L), and *X*_3_ represents the acetic acid adding time. Table 1 shows the coded factor levels and reals values for the variables. According to the response values obtained from these experimental results, a second-order regression equation was generated for the response surface: *Y* = − 459.44 + 151.32 * *X*_1_ + 1.39 * *X*_2_ + 18.90 * *X*_3_ + 0.07 * *X*_1_*X*_2_ + 0.22 * *X*_1_*X*_3_ − 0.09 * *X*_2_*X*_3_ − 14.21 * *X*_1_^2^ − 0.05 * *X*_2_^2^ − 1.81 * *X*_3_^2^. *F* and *P* values indicated the significance of the regression coefficient. The model *F* value of 0.0134 and *P* value of 0.0012 indicated that this model was significant. Through the analysis of *F* value, the importance order of these variables on the butyl acetate production is as follows: pH > acetic acid addition time > supplemented acetic acid concentration. In addition, the quadratic coefficients of *X*_1_^2^ and *X*_3_^2^ were significant (*P* < 0.05), indicating that these variables had considerable effects on the final butyl acetate production. However, the linear coefficient *X*_2_, *X*_3_ and the interaction coefficients of *X*_1_*X*_2_, *X*_1_*X*_3_ and *X*_2_*X*_3_ were not significant in the estimated model with larger *P* values, suggesting that the interaction of *X*_1_*X*_2_, *X*_1_*X*_3_ and *X*_2_*X*_3_ was slight. The three-dimensional plots of response surfaces demonstrated the interaction between these variables and the optimum condition of each variable for the maximum butyl acetate production, which also supported that the interaction coefficients of *X*_1_*X*_2_, *X*_1_*X*_3_ and *X*_2_*X*_3_ were not significant for the final butyl acetate production. As observed in Fig. 1A–C, the predicted maximum butyl acetate production from the response surface model was 7.13 g/L when supplemented acetic acid concentration, acetic acid addition time, and pH were 15.00 g/L, 120 h, and 5.5, respectively.

**Table 1:** The factors and levels of Box–Behnken experiment

Independent variable	Units	Coded variable levels		
		− 1	0	1
pH		5	5.5	6
Acetic acid concentration	(g/L)	10	15	20
Acetic acid addition time	(h)	4	5	6

In order to further verify the influence of optimized conditions obtained from Design Expert software for butyl acetate synthesis by using *C. acetobutylicum* NJ4, the batch fermentation of *C. acetobutylicum* NJ4 with conditions of 15 g/L of supplemented acetic acid, acetic acid addition time at 72 h, and pH of 5.5 was carried out. As shown in Fig. 1D, the actual titer of butyl acetate reached 7.30 g/L with a yield of 0.34 g/g glucose, which was equivalent to the predicted level. Compared with 0.25 g/L of butyl acetate before the process optimization, the optimized titer of butyl acetate was increased by 29.2-fold. In details, when *C. acetobutylicum* NJ4 was first cultured under anaerobic conditions for 72 h, 7.82 g/L of butanol and 2.55 g/L of acetic acid were accumulated. When 15 g/L of acetic acid was exogenously supplemented at 72 h, the synthesis rate of butanol decreased significantly, and butyl acetate synthesis was onset. Under the optimal conditions, *C. acetobutylicum* NJ4 entered the butanol-producing stage at 24 h. The maximum titer of butyl acetate finally reached 7.30 g/L after 96 h, representing the highest butyl acetate production through microbial fermentation process. As stated, the esterification reaction is a reversible process. When the concentration of butyl acetate reached high levels, it will drive the esterification reaction to the reverse direction. With the prolonging of fermentation duration, butyl acetate was slightly hydrolyzed (Fig. 1D). After 144 h, 55.6 g/L of glucose was consumed; meanwhile, 16.12 g/L of acetic acid and 8.39 g/L of butanol also occurred in the fermentation medium.

### Biosynthesis of butyl acetate by using microbial monoculture of *A. succinogenes* 130z (Δ *pflA*) with the supplementation of exogenous butanol

**Fig. 2:**
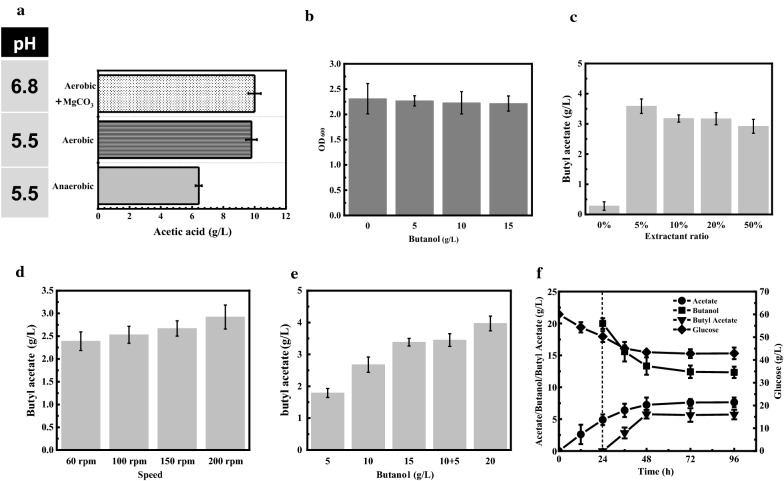
Biosynthesis of butyl acetate by using *A. succinogenes*130z (Δ*pflA*). **A** Acetic acid synthesis of *A. succinogenes*130z (Δ*pflA*) under different conditions. **B** Tolerance of *A. succinogenes*130z (Δ*pflA*) to butanol. **C** Effect of extractant ratio on the final concentration of butyl acetate. **D** Effect of speed on synthesis of butyl acetate. **E** Effect of butanol supplementation concentration on final concentration of butyl acetate synthesis. **F** Fermentation profiles of *A. succinogenes*130z (Δ*pflA*) under optimal conditions

*A. succinogenes* 130z (Δ *pflA*) was genetically constructed by our laboratory, which can be used for acetic acid production due to the deletion of pyruvate formate-lyase activating enzyme (*pflA*) [22]. As known, acetic acid is also one main precursor for butyl acetate synthesis, accordingly, butyl acetate synthesis capability was evaluated by using *A. succinogenes* 130z (Δ *pflA*) based on similar principles as solventogenic *Clostridium* fermentation process. During the fermentation process of *A. succinogenes* 130z (Δ *pflA*), butanol and lipases were exogenously supplemented for butyl acetate synthesis. Meanwhile, butyl acetate was also simultaneously extracted into the organic phase of dodecane to increase the final titer. It should be noticed that *A. succinogenes* 130z (Δ *pflA*) is a facultative strain, and its acetic acid production capability varies significantly under aerobic and anaerobic conditions. Hence, acetic acid production capabilities by using *A. succinogenes* 130z (Δ *pflA*) under different conditions were first investigated. As shown in Fig. 2A, *A. succinogenes* 130z (Δ *pflA*) was capable of producing 10.02 g/L of acetic acid in the presence of oxygen and MgCO_3_, which can maintain the fermentation pH at 6.8 in the batch fermentation process. However, only 6.43 g/L of acetic acid was produced by using *A. succinogenes* 130z (Δ *pflA*) under anaerobic conditions without MgCO_3_ when pH was controlled at 5.5. However, under aerobic conditions, pH did not show any obvious effects on the final acetic acid production by using *A. succinogenes* 130z (Δ *pflA*). For example, 9.95 g/L of acetic acid was still produced under aerobic conditions with pH of 5.5. Taken together, aerobic conditions with pH controlled at 5.5 were adopted for the subsequent fermentations. On the other hand, high butanol concentration would lyse cell membrane and cause toxicity to microbes [7]. Accordingly, butanol toxicity of *A. succinogenes* 130z (Δ *pflA*) was also evaluated. As shown in Fig. 2B, when exogenous butanol (up to 15 g/L) was supplemented into the fermentation broth of *A. succinogenes* 130z (Δ *pflA*), there was almost no difference in the cell growth compared to that without butanol supplementation, indicating that low butanol concentration almost had no effect on the growth of strain 130z Δ *pflA*.

The ratio of extractant to medium directly affects the final butyl acetate concentration. The higher of the extractant proportion, the more butyl acetate can be simultaneously extracted from the aqueous phase. As observed from Fig. 2C, less than 0.5 g/L of butyl acetate was detected in the aqueous phase of the fermentation system without the addition of the extractant of dodecane. When 5% extractant was added into the fermentation broth, butyl acetate concentration in the organic phase was improved to 3 g/L. However, further improvement of extractant ratio to 50% did not enhance the final butyl acetate production. The butyl acetate concentration produced by the aqueous phase was further compared (concentration of butyl acetate in aqueous phase = concentration of butyl acetate in organic phase * ratio of extractant). When the extractant ratio was 50%, the butyl acetate concentration produced by the aqueous phase was the highest, which was 1.46 g/L.

Aeration can facilitate acetic acid production of *A. succinogenes* 130z (Δ *pflA*), which would affect the final butyl acetate production. As shown in Fig. 2D, 2.38 g/L of butyl acetate can be synthesized at 60 rpm. While at 200 rpm, the butyl acetate titer reached 2.92 g/L, which increased by 22.7%. Furthermore, with the increase of butanol supplementation, the butyl acetate titer was also improved. For example, when 5 g/L of exogenous butanol was supplemented, butyl acetate was only 1.79 g/L. When 20 g/L of exogenous butanol was supplemented, the butyl acetate titer could reach 3.97 g/L, which increased by 121.7%. In addition, feeding butanol twice or once with total concentration of 15 g/L had no effects on the final butyl acetate production (Fig. 2E). Taken together, when 20 g/L of butanol was added at 24 h, 5.76 g/L of butyl acetate was synthesized by *A. succinogenes* 130z (Δ *pflA*) at 200 rpm with the yield of 0.35 g/g glucose, representing the first report on butyl acetate production through acetate production process (Fig. 2F, Table 2).

**Table 2:** Comparison of short-chain fatty acid esters production by lipases

Product	Stain	Substrate	Lipase	Titer	Conversion rate	References
Butyl oleate	–	Oleic acid + butanol	Immobilized *R. oryzae* lipase	–	73%	[40]
Butyl lactate	–	Ethyl lactate + butanol	Novozyme 435	–	93.6%	[8]
Butyl butyrate	*Clostridium acetobutylicum*	Glucose + butyric acid	Novozyme 435	5 g/L	0.17 g/g	[19]
Butyl butyrate	*Clostridium tyrobutyricum*	Glucose + butanol	Novozyme 435	34.7 g/L	0.69 g/g	[15]
Butyl butyrate	*Clostridium tyrobutyricum* + *Clostridium beijerinckii*	Glucose	Novozyme 435	5.1 g/L	0.07 g/g	[1]
Butyl butyrate	*E. coli consortium*	Glucose	Novozymes Lipozyme CALB	7.2 g/L	0.12 g/g	[17]
Butyl acetate	*C. acetobutylicum* NJ4	Glucose + acetic acid	Novozyme 435	7.30 g/L	0.34 g/g	This study
Butyl acetate	*A. succinogenes*130z(Δ*pflA*)	Glucose + butanol	Novozyme 435	5.76 g/L	0.35 g/g	This study
Butyl acetate	*C. acetobutylicum* NJ4 + *A. succinogenes*130z(Δ*pflA*)	Glucose	Novozyme 435	2.20 g/L	0.06 g/g	This study
Butyl acetate	*C. acetobutylicum* NJ4 + *A. succinogenes*130z(Δ*pflA*)	Glucose + acetic acid	Novozyme 435	2.86 g/L	0.05 g/g	This study

### Biosynthesis of butyl acetate by using microbial co-culture composed of *C. acetobutylicum* NJ4 and *A. succinogenes* 130z (Δ *pflA*)

**Fig. 3:**
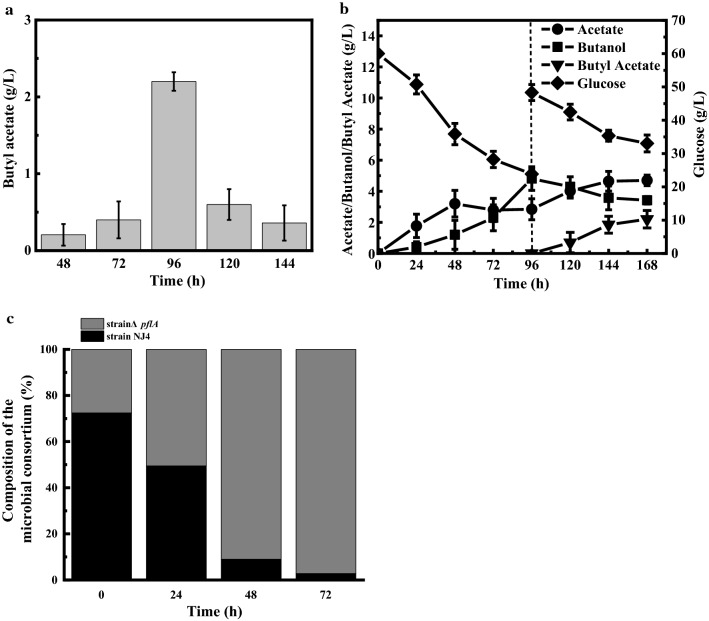
Biosynthesis of butyl acetate by using microbial co-culture system. **A** Effect of mixing time on butyl acetate biosynthesis by using microbial co-culture system. **B** Fermentation profiles of microbial co-culture system under optimal conditions. **C** Changes of community composition during synthesis of butyl acetate by microbial co-culture system under optimum conditions

Different from the above investigated two examples, microbial co-culture system can eliminate the supplementation of exogenous acid or alcohol during the butyl acetate synthesis process, which will reduce the production cost especially at the large scale. Accordingly, the microbial co-culture system composed of *C. acetobutylicum* NJ4 and *A. succinogenes* 130z (Δ *pflA*) was evaluated for butyl acetate production without the supplementation of any acetic acid or butanol. Based on their growth and metabolic characteristics, this microbial co-culture system can be divided into two stages. In the first one, butanol can be specifically synthesized by solventogenic *C. acetobutylicum* NJ4, while in the second one, *A. succinogenes* 130z (Δ *pflA*) can be inoculated, which was mainly responsible for the synthesis of acetic acid. Both butanol and acetic acid can be simultaneously converted into butyl acetate under the esterification of lipases. The inoculation time of *A. succinogenes* 130z (Δ *pflA*) showed significant effects on the final butyl acetate production. As shown in Fig. 3A, when *A. succinogenes* 130z (Δ *pflA*) was inoculated at 48 h, the butyl acetate titer was only 0.2 g/L. However, when *A. succinogenes* 130z (Δ *pflA*) was inoculated at 96 h, the butyl acetate titer reached 2.2 g/L at 168 h, which was almost tenfold higher than that at 48 h (Fig. 3B). When *A. succinogenes* 130z (Δ *pflA*) was inoculated at the late fermentation stage of *C. acetobutylicum* NJ4 (120 h), butyl acetate production decreased.

As shown in Fig. 3B, before the inoculation of *A. succinogenes* 130z (Δ *pflA*), *C. acetobutylicum* NJ4 produced 2.84 g/L of acetic acid and 4.82 g/L of butanol with the consumption of 36.15 g/L of glucose. Once *A. succinogenes* 130z (Δ *pflA*) was inoculated, butyl butyrate was synthesized. With the increase of butyl acetate, butanol concentration decreased, indicating that butanol was simultaneously catalyzed into butyl acetate. Conversely, acetic acid production increased with the synthesis of butyl acetate. The proportion change of bacteria composition in this microbial co-culture system was also analyzed by qPCR (Fig. 3C). As shown in Fig. 3C, at 72 h after the co-cultivation of *A. succinogenes* 130z (Δ *pflA*), the percentage of *C. acetobutylicum* NJ4 in this microbial co-culture system decreased from 72.26% to 2.74%, while the percentage of *A. succinogenes* 130z (Δ *pflA*) increased from 27.54% to 96.26%. *A. succinogenes* 130z (Δ *pflA*) became the dominant strain within this microbial co-culture system at the late-stage fermentation for butyl acetate production. As butanol production by using *C. acetobutylicum* NJ4 was a more complex process than acetic acid production one by using *A. succinogenes* 130z (Δ *pflA*), excess acetic acids in this microbial co-culture system would inhibit the expression of butanol formation genes and microbial metabolism, leading to the decreased cells of *C. acetobutylicum* NJ4 [1]. Moreover, the proportion change of bacteria composition within this microbial co-culture system was also in accordance with the change of metabolic profiles, in which butanol was first synthesized followed by acetic acid synthesis (Fig. 3B).

### Transcriptional analysis of key genes expression levels for butyl acetate synthesis in microbial co-culture system

**Fig. 4:**
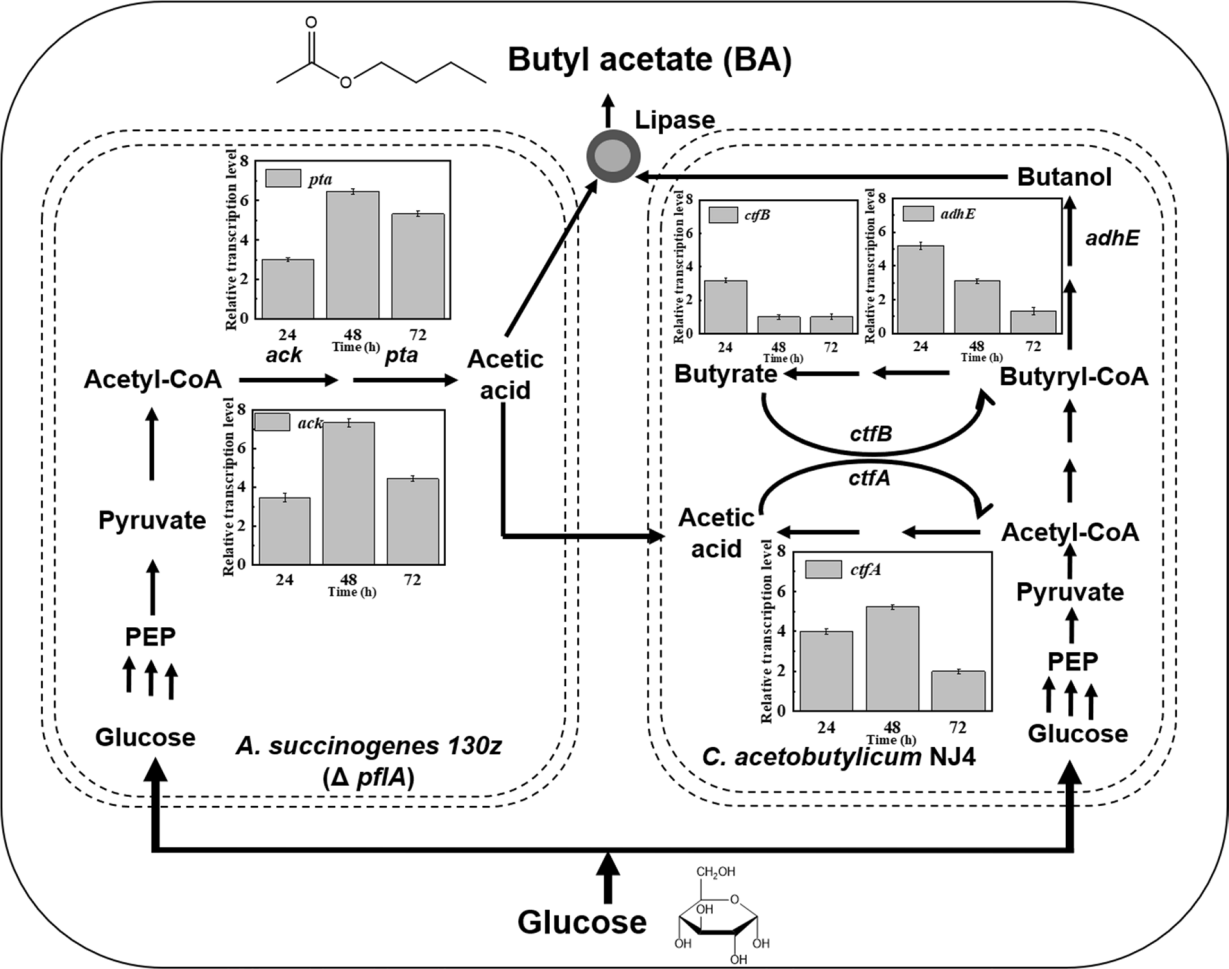
Analysis of key genes expression levels for butyl acetate synthesis in microbial co-culture system. The transcription levels of key genes for acid reassimilation (*ctfA* and *ctfB*) and butanol production (*adhE*) of *C. acetobutylicum* and key genes in acetic acid production (*pta* and *ack*) of *A. succinogenes*130z (Δ*pflA*)

To elaborate the interaction mechanism after these two strain members were co-cultivated, the transcription levels of key genes were analyzed. As observed from Fig. 4, the functional modules of this co-culture system can be divided into two parts. The first is the acetic acid synthetic module within *A. succinogenes* 130z (Δ *pflA*), and the second is the butanol synthetic module within *C. acetobutylicum* NJ4. For the butanol-producing strain of *C. acetobutylicum* NJ4, the expression levels of alcohol/aldehyde dehydrogenase (*adhE*) and CoA transferase B (*ctfB*) genes related to butanol synthesis and butyric acid re-utilization decreased gradually with the increase of the microbial co-cultivation duration (Fig. 4). After co-cultured with *A. succinogenes* 130z (Δ *pflA*), the expression levels of *ctfB* and *adhE* in *C. acetobutylicum* NJ4 showed 5.21- and 3.2-fold increase, respectively, at 24 h compared with those of the microbial monoculture of strain NJ4. However, only 1.32- and 1-fold increase for the expression levels of *adhE* and *ctfB* was observed at 72 h. Different from those of butanol synthetic genes, the expression levels of CoA transferase A (*ctfA*) responsible for acetic acid re-utilization showed a trend of increase first and then decrease [23]. In details, the expression levels of *ctfA* showed fourfold increase at 24 h compared to that of microbial monoculture, and then its expression levels increased to 5.23-fold at 48 h. After 72 h, its expression levels decreased by twofold. The change of *ctfA* expression levels in this microbial co-culture system may be attributed to the high acetic acid production by *A. succinogenes* 130z (Δ *pflA*).

In terms of the acetic acid synthetic module, the transcription levels of key genes *pta* and *ack* for acetic acid production within *A. succinogenes* 130z (Δ *pflA*) showed a similar profile with that of *ctfA* within *C. acetobutylicum* NJ4, both of which increased first and then decreased (Fig. 4). In details, after the microbial co-culture was onset, the expression levels of *pta* and *ack* showed 3- and 3.42-fold increase at 24 h, respectively. With the increase of acetic acid production, the expression levels of *pta* and *ack* genes also increased significantly. For example, the highest increase of 6.45- and 7.32-fold for *pta* and *ack* expression levels was observed at 48 h. As shown in Fig. 3B, the highest acetic acid production also occurred after *A. succinogenes* 130z (Δ *pflA*) was co-cultured with *C. acetobutylicum* NJ4 for 48 h. When the fermentation duration was extended to 72 h, their expression levels decreased to 5.32- and 4.43-fold, respectively (Fig. 4). These indicated that the acetic acid productivity is the highest at 48 h. Taken together, the similar expression profile of *ctfA* in *C. acetobutylicum* NJ4 and *pta* and *ack* in *A. succinogenes* 130z (Δ *pflA*) proved that the acetic acid synthetic module can promote the acetic acid complement pathway for butanol production.

### Improved butyl acetate production by using microbial co-culture composed of *C. acetobutylicum* NJ4 and immobilized *A. succinogenes*130z (Δ*pflA*)

**Fig. 5:**
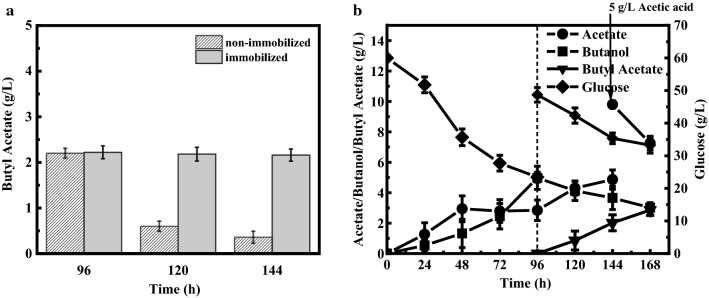
Butyl acetate production by using microbial co-culture system composed of *C. acetobutylicum* NJ4 and immobilized *A. succinogenes*130z (Δ*pflA*). **A** Comparison of butyl acetate synthesis by microbial co-culture systems with immobilized and non-immobilized *A. succinogenes*130z (Δ*pflA*). **B** Synthesis of butyl acetate by adding 5 g/L of acetic acid in co-culture system

Generally, the microbial co-culture system is unstable as the metabolic products in fermentation medium will affect the microbial growth and metabolic activity [27–29] (Fig. 3C). Especially, when *A. succinogenes* 130z (Δ *pflA*) was inoculated into *C. acetobutylicum* NJ4 fermentation medium, the butanol initially produced by strain NJ4 would affect the growth of strain 130z (Δ *pflA*), leading to the instability of strain composition of this microbial co-culture system (Fig. 3B). Material intervened biological fermentation has been proved as an effective method to improve the stability of microbial system. Especially, sodium alginate embedding technology has been widely used in microbial co-culture systems, which could effectively improve the stability of microbial co-culture systems [28, 29]. Sodium alginate and calcium ions can be crosslinked to form insoluble gel, in which cells can be immobilized in gel beads. Furthermore, alginate gel beads can reduce the solvent damage to cell membrane [30, 31]. Accordingly, the embedded *A. succinogenes*130z (Δ*pflA*) in sodium alginate was inoculated into this microbial co-culture system to further improve the final butyl acetate production. As shown in Fig. 5A, the inoculation time of embedded cells of *A. succinogenes*130z (Δ *pflA*) will not affect the final butyl acetate production; however, the inoculation time of free cells of *A. succinogenes*130z (Δ *pflA*) showed significant effects on the butyl acetate production. For instance, the final butyl acetate production was maintained at around 2.18 g/L, no matter when embedded cells of *A. succinogenes*130z (Δ *pflA*) was inoculated at 96, 120, or 144 h. Instead, comparable butyl acetate production only occurred at 96 h when free cells of *A. succinogenes*130z (Δ *pflA*) were inoculated. When free cells of *A. succinogenes*130z (Δ *pflA*) were inoculated at 120 or 144 h, butyl acetate production was below 0.6 g/L (Fig. 5A). The reason could be that higher amount of butanol (12.2 g/L and 14.5 g/L) was produced by strain NJ4 at 120 or 144 h (data not shown here), and this high initial butanol concentration will affect the bacterial growth and metabolic activity of strain 130z (Δ *pflA*).

To enhance the final butyl acetate concentration by using this microbial co-culture system with embedded cells of *A. succinogenes*130z (Δ *pflA*), 5 g/L of acetic acid was further supplemented exogenously to drive the esterification process toward synthetic rather than hydrolytic sides (Fig. 5B). In details, when *C. acetobutylicum* NJ4 was cultured for 96 h, it produced 2.84 g/L of acetic acid and 4.98 g/L of butanol (Fig. 5B). At this time, embedded cells of *A. succinogenes*130z (Δ*pflA*) were inoculated. 2.02 g/L of butyl acetate was synthesized after total 144 h. Further extension of the fermentation duration cannot improve the butyl acetate production, indicating the esterification reached the equilibrium state. When 5 g/L of acetic acid was fed, the maximum 2.86 g/L of butyl acetate was obtained, which was 30% higher than that of the microbial co-culture system without acetic acid.

## Discussion

Three different fermentation strategies for the one-pot butyl acetate production have been designed in this study. Within these three systems, microbial monoculture system always gave higher butyl acetate titer than that of co-culture system (Figs. 1D, 2E and 3B). These findings were in consistency with those of other short-chain fatty acid esters production, mainly butyl butyrate production systems [15, 17]. For example, the highest butyl butyrate production occurred in butyrate production system (34.7 g/L), followed by butanol production system (22.4 g/L) and microbial co-culture system (7.2 g/L) [15, 17, 20].

As known, lipases possess both hydrolytic and synthetic activities [15, 19]. Higher concentrations of acids or alcohols will help facilitate lipases toward synthetic rather than hydrolytic sides in microbial monoculture system [19]. For instance, Xin et al. fed total 7.8 g/L of butyrate (at 0, 48 and 72 h) to improve the butyl butyrate production to 22.4 g/L by using solventogenic *Clostridium* sp. BOH3 [20]. It should be noticed that strain BOH3 also indigenously generated some amount of butyrate [20]. Zhang et al. maintained butanol concentration at 10 g/L during 168 h fermentation process by using *C. tyrobutyricum*, and 34.7 g/L of butyl butyrate was produced [15]. Similarly, the improvement of acetic acid or butanol concentration in butanol or acetic acid production system also helped improve the final butyl acetate production [32]. Recently, a genetically modified *C. beijerinckii* has been constructed for butyl acetate production, which gave the highest 5.57 g/L of butyl acetate from 38.2 g/L of glucose within 48 h; however, 5 g/L of acetic acid was still needed to be supplemented to boost the final butyl acetate production [7]. The lower concentration of butyl acetate compared to butyl butyrate production system could be attributed to the lower equilibrium constant of lipases in butyl acetate production systems [15]. Further studies to adopt more efficient butanol or acetic acid production system and improvement of lipases equilibrium constant toward synthetic sides should be carried out to improve the final butyl acetate production efficiency [15, 19]. It should be noticed that the supplementation of higher amount of precursors would significantly increase the production cost when using microbial monoculture system especially for the large-scale production of short-chain esters, as the typical reaction ratio of acid and alcohol was 1:1. Alternatively, microbial co-culture system could solve this problem efficiently through the construction of both acid- and alcohol-producing microbial members although the production level is lower than microbial mono-culture system.

Recently, microbial co-culture system has been widely used to synthesize complex structure chemicals, such as plant derived natural products [33, 34]. In terms of short-chain fatty acid esters, microbial co-culture system shows advantages in the elimination of alcohol or acid supplementation [1, 17]. For example, a *Clostridium*–*Clostridium* co-culture system has been designed for esters mainly butyl butyrate production, which was the first study reporting microbial co-culture for the production of short-chain fatty acid esters [1]. By adopting this strategy, 5.1 g/L of butyl butyrate could be produced without the addition of exogenous precursors, representing a more cost effective strategy to produce esters [1]. Furthermore, a cognate *E. coli* consortium was also constructed to produce 7.2 g/L of butyl butyrate without the exogenous addition of butanol or butyrate [17]. To the best of our knowledge, this is the highest titer and yield of butyl butyrate production by using microbial co-culture system reported to date [17]. Inspired by the successes of these examples, we constructed a *Clostridium* and *Actinobacillus* co-culture system in this study, which finally produced 2.86 g/L of butyl acetate, representing the first study on butyl acetate production by using microbial co-culture system. The success of this system further proved and paved a new way for the biotechnological production of other short-chain fatty acid esters, such as acetyl acetate and butyl lactate. Further studies to improve the stability of strain members and optimize the fermentation conditions should be investigated to improve the final esters production efficiency.

## Conclusion

In this study, microbial mono- and co-culture systems for butyl acetate biosynthesis were successfully constructed. The highest 7.30 g/L of butyl acetate with a yield of 0.34 g/g glucose can be produced by using microbial monoculture of *C. acetobutylicum* NJ4 with the supplementation of exogenous acetic acid after the process optimization. Moreover, the highest 2.86 g/L of butyl acetate was produced by using microbial co-culture system composed of *C. acetobutylicum* NJ4 and *A. succinogenes* 130z (Δ *pflA*) with the elimination of butanol and acetic acid supplementation. To the best of our knowledge, these represent the first studies regarding butyl acetate production through microbial mono- and co-culture fermentation systems. During these processes, although lipases are the most widely used enzymes for the esterification of carboxylic acids with alcohols, their cost remains a problem. To tackle this obstacle, future studies should focus on overproducing recombinant lipases for selective ester biosynthesis. Moreover, the challenge of different oxygen requirements for acetate and butanol biosynthesis needs to be addressed to achieve a higher yield of butyl acetate.

## Materials and methods

### Strains and media

*C. acetobutylicum* NJ4 was isolated and stored by our lab [23, 24]. *A. succinogenes* 130z (Δ*pflA*) was obtained by knocking out *pflA* from *A. succinogenes* 130z (ATCC 55618), which can efficiently produce acetic acid from glucose [22]. The P1 fermentation medium contains 0.75 g/L of KH_2_PO_4_, 0.75 g/L of K_2_HPO_4_, 4.585 g/L of 2-[[2-hydroxy-1,1-bis(hydroxymethyl)ethyl]amino]ethanesulfonic acid, and 5 g/L of yeast extract. 1 mL of Na_2_SeO_3_–Na_2_WO_4_ solution [35], 1 mL of trace element solution [20], 10 mL of salt solution [35], and 10 mg of resazurin (oxygen indicator) were added into 1 L medium, respectively. In addition, 0.024 g/L of l(+)-cysteine was added as reductants under N_2_. The medium (36 mL) and 600 g/L glucose concentrate (4 mL) were dispensed into 100 mL of serum bottle with nitrogen purged and then autoclaved at 121 °C for 15 min [23].

*C. acetobutylicum* NJ4 was cultivated in P1 medium. *A. succinogenes* 130z (Δ*pflA*) was cultivated in medium containing 10 g/L of yeast extract, 1.36 g/L of NaAc, 0.3 g/L of Na_2_HPO_4_·12H_2_O, 1.6 g/L of Na_2_HPO_4_·2H_2_O, 3 g/L of K_2_HPO_4_, 1 g/L of NaCl, 0.2 g/L of MgCl_2_·6H_2_O, 0.2 g/L of CaCl_2_, and 7.5 g/L of corn steep liquor. The microbial consortium was cultivated in P1 medium with 7.5 g/L of corn steep liquor [22, 23].

### Serum bottle fermentation using microbial monoculture and co-culture

For microbial monoculture of *C. acetobutylicum* NJ4, 1 mL of inoculum was added into 40 mL of medium with 60 g/L of glucose. The fermentation batches were incubated at 37 °C with 200 rpm. During the fermentation process, pH was adjusted to 5.5 by using 3 M sodium hydroxide solution. Concentrations of glucose, acetic acid, butanol, and butyl acetate in the sample were determined every 24 h. Each experiment was performed in triplicates.

For microbial monoculture of *A. succinogenes* 130z (Δ*pflA*), 5 mL of inoculum was added into 50 mL medium with 60 g/L of glucose. Besides, during the fermentation process, pH was adjusted to 5.5 by using 3 M sodium hydroxide solution. The fermentation batches were performed at 37 °C with 200 rpm. Concentrations of glucose, acetic acid, butanol, and butyl acetate in the sample were measured every 24 h during the fermentation process. The 50% extraction agent dodecane was added when 100 U/mL lipases were added. Each experiment was performed in triplicates.

For the microbial co-culture fermentation process, *C. acetobutylicum* NJ4 was first inoculated with 60 g/L of glucose as the carbon source under anaerobic conditions at 37 °C, and medium pH was controlled at 5.5 by using 3 M NaOH solution. The inoculation ratio (1:5) has been optimized (data not shown). Then the seed inoculum of *A. succinogenes* 130z (Δ*pflA*) was then added [22]. At the same time, 100 U/mL of lipase and 50% dodecane were added. Then 30 g/L glucose was added to the co-culture medium and the medium pH was still controlled at 5.5. During the fermentation process, concentrations of glucose, acetate, butanol, and butyl acetate in the sample were determined every 24 h.

### Optimization of fermentation conditions

For microbial cultivation of *C. acetobutylicum* NJ4, three factors, including pH, acetic acid supplemental concentration, and acetic acid addition time, were found to have great influence on butyl acetate production. To optimize the fermentation process, the RSM was further applied. Acetic acid supplemental concentration, pH, and acetic acid addition time were independent variables, while butyl acetate concentration was the dependent variable (Table 1). A set of 17 experiments was designed by using the statistical software Design Expert 10. Each experiment was carried out in triplicates. The relationship between dependent and independent variables is explained through the following second order polynomial equation:

1 $$ Y =\,\alpha_{0} + \alpha_{1} X_{1} + \alpha_{2} X_{2} + \alpha_{3} X_{3} + \alpha_{12} X_{1} X_{2} + \alpha_{13} X_{1} X_{3} + \alpha_{23} X_{2} X_{3} + \alpha{_{11} X_{1}}^{2} + \alpha{_{22} X_{2}}^{2} + \alpha_{33} X{_{3}}^{2} $$

where *Y* is predicted response (butyl acetate concentration); *X*_1_, *X*_2_, and *X*_3_ are independent variables (pH, acetic acid supplemental concentration and acetic acid addition time); *α*_0_ is offset term; *α*_1_, *α*_2_, and *α*_3_ are linear effects; *α*_12_, *α*_13_, and *α*_23_ are squared effects; and *α*_11_, *α*_22_, and *α*_33_ are interaction terms. Analysis of variance (ANOVA) was used to perform statistical analysis of the model [35]. For microbial monoculture of *A. succinogenes* 130z (Δ*pflA*), the influence of several single factors, such as extracting agent, lipase supplemental level, speed, and butanol concentration, on the butyl acetate production was explored. Then, the optimized monoculture conditions were used to produce butyl acetate by microbial co-culture. For the microbial co-culture system, the inoculation time of co-culture was optimized and the titer of butyl acetate increased by immobilization.

### Analysis of relative transcriptional levels

Total RNAs from different samples of co-culture were extracted with FastPure Cell/Tissue Total RNA Isolation Kit (Vazyme, Nanjing, China). The DNA was removed and then the RNAs were reverse transcribed to complementary DNA (cDNA) by using the 5 × HiScript II qRT SuperMix II (Vazyme, Nanjing, China). The cDNA was used as a template, and the quantitative real-time polymerase chain reaction (qPCR) assay was performed by using ChamQTM SYBR® qPCR Master Mix (High ROX Premixed, Vazyme, Nanjing, China) in Applied Biosystems (StepOne Plus) to quantify the transcription levels of related genes. The primers used in this study are listed in Table 3. The expression level of target gene was calculated by the method of 2^−ΔΔCT^ [36]. The 16S rRNA gene was used to standardize the mRNA levels. Since 16S ∆*pflA* (amplification fragment) and 16S NJ4 (amplification fragment) are expressed with 6 and 11 copies in the genomes of *A. succinogenes* 130z (Δ*pflA*) and *C. acetobutylicum* NJ4, respectively, the abundance of each strain in the co-culture system was determined by Eqs. (2) and (3) [37, 38]:

2 $$ {\text{Abundance}}\;{\text{of}}\;\Delta pflA = \frac{{16S\Delta pflA\;{\text{copy}}\;{\text{number}}/6}}{{16S\Delta pflA\;{\text{copy}}\;{\text{number}}/6 + 16S\;{\text{NJ}}4\;{\text{copy}}\;{\text{number}}/11}}, $$

3 $$ {\text{Abundance}}\;{\text{of}}\;{\text{NJ}}4 = \frac{{16S\;{\text{NJ}}4\;{\text{copy}}\;{\text{number}}/11}}{{16S\Delta pflA\;{\text{copy}}\;{\text{number}}/6 + 16S\;{\text{NJ}}4\;{\text{copy}}\;{\text{number}}/11}}. $$

**Table 3:** Primers used in this study

Primer	Sequence
r16s 130z	GCTTTCCATGCTGACGAGTG/GTCGGCTTGGTAGGCCTTTA
r16s NJ4	GGCAGCAGTGGGGAATATTG/CGCCTACACATCCTTTACGC
*pta*	TATTGGTGTACGGCGACTGT/GCGATACGGGTTGCTTCTTT
*ack*	CAACCCTGCCCACTTAATCG/ACCTAAACGTTTTGCCGCTT
*adhe*	ACGGACTAGCACTAGAGGCAAT/CCATAGTTGAAGCGTGAGCCAT
*ctfA*	CGGATCTGGCTTAGGTGGTGTA/TGCTACATCGGCTGTAAGAGGT
*ctfB*	ATGCTCTCTGGTATGGGTGGAG/TTGCTTGAGACTTTGCCGTGAG

### Sodium alginate immobilization

2.0 g of sodium alginate was dissolved in 100 mL DI water, which was then autoclaved at 120 °C for 15 min. *A. succinogenes* 130z (Δ *pflA*) in logarithmic growth phase was evenly mixed with sodium alginate solution with the ratio of 1:10. The mixture of sodium alginate (10 mL) and strain 130z Δ *pflA* (1 mL) was aspirated with syringe; 20 g/L of CaCl_2_ solution (80 drops/min) was dripped by drip. Immediately, smooth gel beads were formed, which was then hardened at ambient temperature for 30 min and filtered out of the gel beads. After washed with sterile water, the gel beads were filtered out again. After sterilized medium was washed, the surface water was removed with absorbent paper [29].

### Analytical methods

Concentrations of acetic acid and glucose were measured by high-performance liquid chromatography (HPLC; UltiMate 3000 HPLC system; Dionex, Sunnyvale, CA) using an ion-exchange chromatographic column (Bio-Rad Aminex HPX‐87H column) at a wavelength of 215 nm on a UVD 170U ultraviolet detector [24]. Butanol and butyl acetate were detected by gas chromatography (GC-2010, Shimadzu Scientific Instruments, Japan) equipped with an InterCap WAX column (0.25 mm × 30 m, GL Sciences Inc., Japan) and a flame ionization detector (FID) [7]. All samples were centrifuged at 12,000 g for 5 min; then 50 μL HCl (2 M) was added in 950 mL of samples. Isobutanol was used as internal standard. The total volume of biogas production was measured on-line through a mass flow controller, a mass flow meter (CS200-A,C,D MFC/MFM, Sevenstar, China), and a gas flow accumulator (D08-8C, Sevenstar, China) [39].

## Supplementary Information

**Additional file 1: Figure S1.** Effect of pH on final butyl acetate production by *C. acetobutylicum* NJ4. **Figure S2.** Effect of acetic acid addition time on final butyl acetate production by *C. acetobutylicum* NJ4. **Figure S3.** Effect of acetic acid addition concentration on final butyl acetate production by *C. acetobutylicum* NJ4.

## Data Availability

All data generated and analyzed in this study are included in this published article.
