# Supplementary material for: Comprehensive evaluation for the one-pot biosynthesis of butyl acetate by using microbial mono- and co-cultures
Source: Biotechnol Biofuels. 2021 Oct 16;14:203. doi: 10.1186/s13068-021-02053-2 (PMC8520270; doi:10.1186/s13068-021-02053-2)
Supplement: Supplementary file 1 — Additional file 1: Figure S1. Effect of pH on final butyl acetate production by C. acetobutylicum NJ4. Figure S2. Effect of acetic acid addition time on final butyl acetate production by C. acetobutylicum NJ4. Figure S3. Effect of acetic acid addition concentration on final butyl acetate production by C. acetobutylicum NJ4. [file 13068_2021_2053_MOESM1_ESM.doc]

**Additional materials of**

**Comprehensive evaluation for the one-pot biosynthesis of butyl-acetate by using microbial mono and co-cultures**

Yang Lv^a^, Yujia Jiang^a^*, Jiasheng Lu^a^, Hao Gao^a^, Weiliang Dong^a,b^, Jie Zhou^a^,

Wenming Zhang^a,b*^, Fengxue Xin^a,b*^, Min Jiang^a,b^

a State Key Laboratory of Materials-Oriented Chemical Engineering,

College of Biotechnology and Pharmaceutical Engineering,

Nanjing Tech University, Nanjing, 211800, P.R. China

b Jiangsu National Synergetic Innovation Center for Advanced Materials, Nanjing Tech University, Nanjing, 211800, P.R. China

*Corresponding authors at: State Key Laboratory of Materials-Oriented Chemical

Engineering, College of Biotechnology and Pharmaceutical Engineering, Nanjing

Tech University, Puzhu South Road 30#, Nanjing 211800, P. R. China.

E-mail addresses:

zhangwm@njtech.edu.cn (W.M. Zhang), xinfengxue@njtech.edu.cn (F.X. Xin),

jiangyujia@njtech.edu.cn (J.Y. Jiang)

**Figure S1** Effect of pH on final butyl acetate production by *C. acetobutylicum* NJ4

**Figure S2** Effect of acetic acid addition time on final butyl acetate production by *C. acetobutylicum* NJ4

**Figure S3** Effect of acetic acid addition concentration on final butyl acetate production by *C. acetobutylicum* NJ4
